# Dietary exogenous microRNAs in the gut: biological effects, mechanistic evidence, and controversies

**DOI:** 10.3389/fnut.2026.1974997

**Published:** 2026-10-16

**Authors:** Andrea Méndez-García, Marely Abigail García-Mendoza, Paulina Pérez-Martínez, Marcela Figueroa-Ramírez, Antara Banerjee, Surajit Pathak, Asim K. Duttaroy, Sujay Paul

**Affiliations:** 1 Tecnologico de Monterrey, School of Engineering and Sciences, San Pablo, Queretaro, Mexico; 2 Tecnologico de Monterrey, School of Engineering and Sciences, Ciudad de Mexico, Mexico; 3 Faculty of Allied Health Sciences, Chettinad Academy of Research and Education, Chettinad Hospital and Research Institute (CHRI), Chennai, India; 4 Department of Nutrition, Institute of Medical Sciences, Faculty of Medicine, University of Oslo, Oslo, Norway

**Keywords:** cross-kingdom communication, cross-species communication, dietary exogenous miRNAs, extracellular vesicles, gut microbiota, intestinal homeostasis

## Abstract

Dietary exogenous microRNAs (miRNAs) have emerged as potential mediators of interorganismal communication, with growing interest in their ability to influence gastrointestinal physiology and the gut microbiota. These molecules encompass plant-derived miRNAs implicated in cross-kingdom regulation, animal-derived dietary miRNAs involved in cross-species communication, and maternal milk miRNAs that may participate in mother-to-offspring molecular crosstalk. A proportion of dietary miRNAs are associated with extracellular vesicles or other protective complexes, which may enhance their stability during food processing and gastrointestinal transit and facilitate interactions with intestinal epithelial cells, immune cells, and microbial communities. This review critically evaluates recent computational, detection-based, *in vitro*, *in vivo*, and human observational evidence concerning the gastrointestinal stability, uptake, target engagement, and biological activity of dietary exogenous miRNAs. Experimental studies have reported effects on inflammatory signaling, epithelial barrier integrity, macrophage polarization, intestinal development, tumor-associated pathways, and gut microbial composition and gene expression. However, the strength of this evidence varies considerably. In particular, detection of dietary miRNAs in food, extracellular vesicles, feces, or recipient tissues does not by itself establish functional target regulation, while studies using purified vesicles, synthetic miRNA mimics, or engineered delivery systems may involve exposures that differ substantially from normal dietary intake. Moreover, biological effects observed following whole-vesicle administration cannot always be attributed specifically to their miRNA cargo. Collectively, current evidence provides stronger support for the biological plausibility of local gastrointestinal effects than for widespread systemic regulation after dietary exposure. Further studies using physiologically relevant doses, rigorous miRNA-specific loss-of-function approaches, direct target validation, standardized analytical methods, and controlled human interventions will be essential to establish the functional significance and translational potential of dietary exogenous miRNAs.

## 1 Introduction

MicroRNAs (miRNAs) are small (~20–24 nt), non-coding, single-stranded RNA molecules that interact with complementary sequences of diverse messenger RNAs (mRNAs) to regulate their expression (1–3). Produced by mammals (4), plants (5–7), and even parasites (8) and viruses (9), these transcripts have been discovered to modulate a wide range of metabolic pathways within the host, with endogenous modulation constituting the most extensively studied mechanism. In fact, miRNAs have been recognized as among the most biologically relevant bioactive molecules in recent years (10). Interestingly, dietary-derived miRNAs have been detected in the serum, tissues, organs, and feces of animals, including humans (11). These miRNAs, termed dietary exogenous miRNAs, are primarily incorporated into the recipient organism via oral consumption (12). The concept of dietary exogenous miRNAs emerged relatively recently. Over a decade ago, Zhang et al. (13) documented the presence of orally acquired exogenous plant miRNAs in the sera and tissues of various animals. More intriguingly, functional studies conducted in cells and mice showed that miR168a, highly abundant in rice, could bind to human/mouse low-density lipoprotein receptor adapter protein 1 (*LDLRAP1*) and inhibit its expression in the liver, consequently impairing hepatic LDL clearance (13). Although multiple subsequent investigations have indicated that external miRNAs can influence the expression of selected host and even bacterial genes, the precise mechanism and physiological scope remain a matter of debate (14). Nonetheless, a recent and comprehensive appraisal in this field has cast doubt on the capacity of plant-derived exogenous miRNAs to mediate cross-kingdom RNA interference (15), underscoring that the debate is far from settled. Bearing this in mind, the accumulated evidence of circulating food-derived miRNAs in heterotrophic organisms opens a novel paradigm for exploring cross-kingdom molecular interactions and, potentially, therapeutic approaches deserving further scrutiny.

The biogenesis of microRNAs is contingent upon the host organism. In plants, broadly, microRNA-encoding genes are transcribed into pri-miRNAs by RNA polymerase II, which then adds a 5′ cap and a 3′ poly(A) tail for stability. Afterward, the transcript, sequestered within the Dicing Bodies (D-bodies) of the nucleus, is cleaved by the RNase III Dicer-like 1 (DCL1) twice. The resulting miRNA/miRNA* is methylated to confer protection against nuclease degradation, which is then exported to the cytoplasm via HASTY (HST) and loaded onto AGO1 while the miRNA* strand is discarded (16, 17). By contrast, in animals, RNA polymerase II transcribes microRNA-encoding genes into pri-miRNAs, bearing a 5′ cap and a 3′ poly (A) tail. Then, the microprocessor DGCR8 and the Drosha enzyme cleave the pri-miRNA stem-base, releasing a hairpin intermediate known as pre-miRNA, which is then shuttled to the cytoplasm by Exportin-5 (XPO5) and Ran-GTP. Once there, RNase III Dicer generates the miRNA/miRNA* duplex by cleavage and the guide strand is loaded into Argonaute RISC Catalytic Component 2 (AGO2) (18).

The main differences between plant and animal miRNA biosynthesis concern subcellular localization. In plants, most of the process occurs in the nucleus, whereas in animals, these steps are distributed evenly between the nucleus and the cytoplasm. Moreover, in plants, a single enzyme, DCL1, performs both principal cleavages, while animals rely on two distinct enzymes to accomplish the same task (19). More interestingly, plant miRNAs exhibit higher stability due to their 3′ end 2′-O-methylation, which prevents 3′ uridylation and 3′ truncation (17). The functional remit of miRNAs likewise diverges markedly at each level. For instance, in plants, they govern plant development, plasticity, biotic and abiotic stress responses, and symbiotic interactions with the environment (20). On the other hand, in animals, they regulate nearly the entirety of biological processes, including apoptosis, cell differentiation, inflammation, cell growth and proliferation, insulin secretion, cholesterol metabolism, and stress responses, among numerous others (21–24).

MiRNAs regulate the expression of floating cytoplasmic mRNA through sequence-specific, direct interactions, thereby mediating post-transcriptional gene regulation (25). This regulatory activity occurs in virtually every cell type of an organism, as they have been found expressed in the brain (26), heart (27), liver (28), skeletal muscle, adipose tissue (29), intestine (30), and circulating in blood (31), milk (32), and saliva (33). As a matter of fact, studies have revealed pronounced tissue- and condition-specific expression patterns of miRNAs, indicating that these ncRNAs are transcribed directly in specific tissues and at the moment when their activity is required (34).

When exploring cross-kingdom regulation, the interplay among host, tissue, cell, miRNA, and mRNA naturally acquires layers of complexity. The proposed pathway begins with the dietary intake of plant-derived foods containing miRNAs encapsulated within EV-like nanoparticles. During gastrointestinal transit, these vesicles encounter acidic conditions, digestive enzymes, and RNases, which may influence the stability and availability of their miRNA cargo. Within the intestinal lumen, EV-like nanoparticles may interact with intestinal epithelial cells and the gut microbiota, potentially contributing to gene regulation, modulation of signaling pathways, intestinal barrier homeostasis, immune modulation, and microbiota composition. A fraction of these particles or their miRNA cargo may potentially reach the circulation, whereas unabsorbed EV-like nanoparticles and miRNAs may ultimately be excreted in feces (Figure 1). Indeed, a firmly established mechanistic route from exogenous miRNA intake to intestinal uptake remains unclear. The most widely accepted process by which dietary exogenous miRNAs enter and prevail inside a complex organism is through their encapsulation in extracellular vesicles (EVs). These vehicles, generally 40 to 100 nm in diameter but capable of reaching up to 400 nm, are released by diverse cell types, including those of edible plants (35). EVs confer stability upon their miRNA cargo by acting as a physical shield against degrading enzymes, extreme pH levels, temperature fluctuations, and even immunogenic mechanisms (36). It is precisely their heterogeneous composition that enables them to traverse the digestive tract largely intact. Their components include proteins, nucleic acids, sugars, and lipids. In plants, this composition is additionally dominated by phosphatidic acid, galactosyldiacyl-glycerol, phosphatidylethanolamine, monogalactosyl-diacylglycerol, and phosphatidyl-choline. Additionally, their negative zeta-potential confers great stability upon contact with other organisms. It is because of this pronounced structural heterogeneity that their biogenesis diverges into multiple routes, including the multivesicular body and the exocyst-positive organelle pathways (15, 35, 37). EVs carrying dietary exogenous miRNAs have been shown to withstand cooking and digestion processes, as corroborated by *in vitro* emulation of these processes (38). Likewise, dietary miRNAs have been reported in animal and human feces following consumption of miRNA-rich foods.

**Figure 1:**
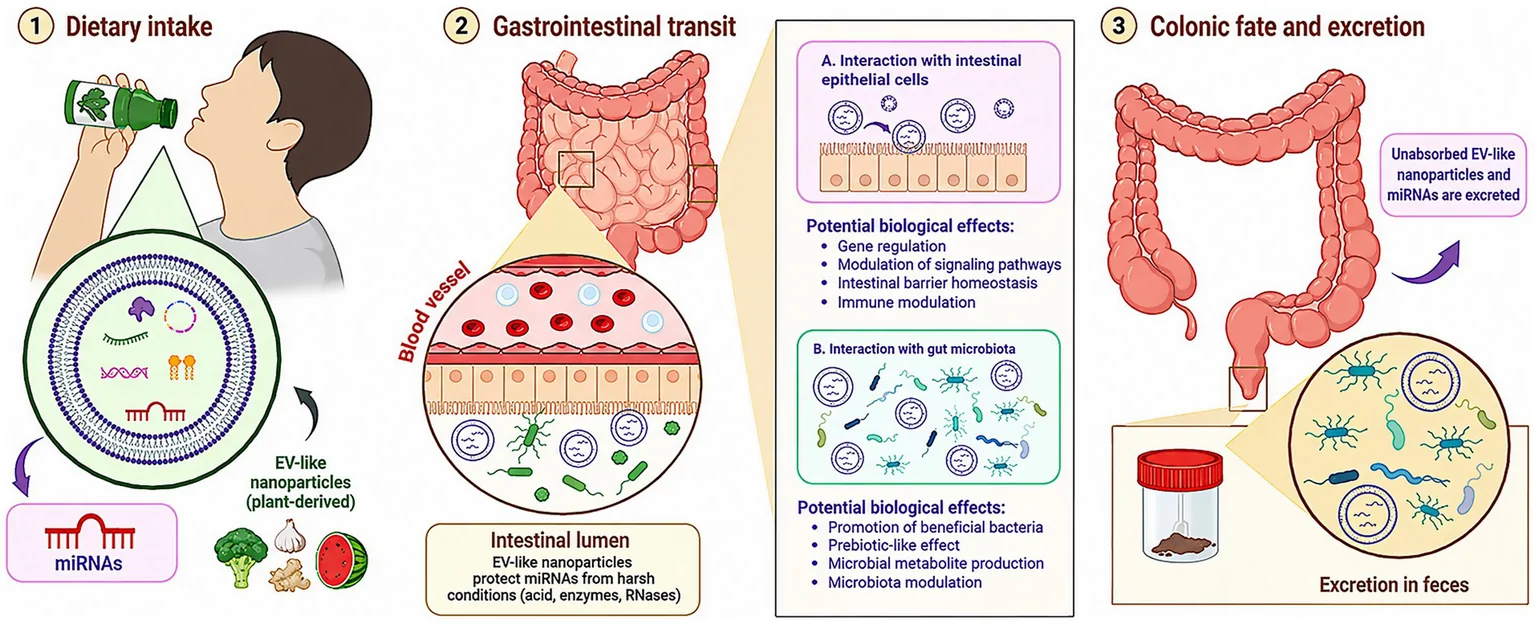
Proposed gastrointestinal processing and biological fate of dietary exogenous miRNAs associated with plant-derived extracellular vesicles. Dietary exogenous miRNAs encapsulated within EVs remain protected throughout gastrointestinal transit and participate in host-microbiota interactions before their digestive excretion. **(1)** Following dietary intake, exogenous miRNAs enter the digestive tract, where the vesicular lipid bilayer protects the encapsulated cargo from acidic pH, digestive enzymes, and RNases. Current evidence suggests that EVs, which can also pack proteins, DNA, lipids, and other types of RNA **(2)** can be internalized by intestinal epithelial and immune cells, where they may contribute to host gene regulation, signaling pathway modulation, immune homeostasis, and intestinal barrier function. EV-associated miRNAs may also interact with members of the gut microbiota, potentially influencing bacterial growth, microbial metabolism, and community composition. **(3)** A proportion of EV-like nanoparticles and their associated miRNAs remain unabsorbed and are ultimately excreted in feces. Image created in BioRender.com.

Several investigators have suggested that orally acquired miRNA-laden EVs are able to modulate signaling pathways in mammalian cells. Although evidence bearing on gastrointestinal miRNA absorption remains contradictory (39), circulating plasma miRNAs of dietary origin have been minimally documented (40). The proposed pathway posits that, once within the gastrointestinal tract, EVs-encapsulated exogenous dietary miRNAs are absorbed by intestinal epithelial cells, macrophages, and the gut microbiota. Most likely, intestinal cells are thought to internalize these EVs via caveolin-mediated endocytosis and macropinocytosis, whereas gut microbiota appear to undergo a lipid-mediated selection process, allowing the vesicular cargo to regulate microbial composition, metabolism, growth, and localization (41).

An ongoing, unresolved debate persists regarding the extent to which dietary miRNAs regulate genes from other kingdoms, as the available literature exhibits notable inconsistencies and reproducibility concerns (38). Some factors that might account for these disparities include variability in experimental methodology, a low proportion of miRNAs surviving the digestive process, and impaired gastrointestinal miRNA absorption (42, 43). Notwithstanding, the reported results in a wide number of studies have opened a discussion worth pursuing, but more importantly, they raise the prospect of formalizing low-risk therapeutic strategies where food-derived, orally administered miRNAs occupy a central role, becoming a potential avenue for further investigation.

This review critically examines recent evidence on exogenous dietary miRNAs originating from different biological sources and acting through distinct modes of interorganismal communication. These include plant-derived dietary miRNAs investigated in the context of cross-kingdom regulation, animal-derived dietary miRNAs associated with cross-species communication, and breast milk miRNAs implicated in maternal–offspring intraspecies crosstalk. Despite their distinct origins, these miRNAs share common challenges related to gastrointestinal stability, intestinal uptake, target engagement, and functional bioavailability. Relevant literature was identified through searches of PubMed, Scopus, and Web of Science, with Google Scholar used as a supplementary source. Searches combined terms related to dietary and exogenous miRNAs with gastrointestinal function and their biological origin, including “dietary miRNA,” “food-derived miRNA,” “xenomiR,” “plant miRNA,” “cross-kingdom miRNA,” “cross-species miRNA,” “milk extracellular vesicle miRNA,” “bovine milk miRNA,” “breast milk miRNA,” and “maternal milk miRNA,” together with terms such as “gut,” “intestinal,” “gastrointestinal,” “microbiota,” and “intestinal homeostasis.” The review primarily considered peer-reviewed original research articles published between 2021 and 2026. Eligible studies included computational, *in vitro*, *in vivo*, human observational, and other experimentally relevant investigations evaluating the gastrointestinal stability, uptake, molecular interactions, biological effects, or microbiota-associated activity of food-derived miRNAs. Studies unrelated to gastrointestinal biology, as well as reviews, editorials, commentaries, and other non-original research publications, were excluded from the primary evidence synthesis. No journal impact factor threshold was applied, and studies were evaluated instead according to their experimental design, mechanistic depth, physiological relevance, and level of functional validation. The final literature search was conducted on August 16, 2026. This framework was designed to provide a critical and up-to-date synthesis of the available evidence while distinguishing computational or associative observations from direct mechanistic and functional evidence and identifying the principal gaps that continue to limit interpretation of dietary miRNA-mediated communication.

## 2 Recent research on the impact and applications of exogenous dietary miRNAs in metabolism and intestinal health

### 2.1 Plant-derived miRNAs conducting cross-kingdom communication

The majority of studies of food-derived miRNAs have been conducted on plant matrices, while other sources remain comparatively under-investigated in the gastrointestinal context. This asymmetry is therefore reflected in the proportion of the studies discussed in this review, and it should be interpreted as a reflection of the available published evidence rather than of biological importance.

The general workflow for the discovery of novel plant-derived miRNAs that might have the potential to regulate commences with RNA extraction from edible plants, which are commonly pretreated with heat, enzymatic, or mechanical forces to test miRNA stability after these cooking- and digestive-related processes. In fact, some research has shown that certain miRNAs can be detected after thermal processing, indicating that cooking does not compromise the structural stability or functionality of a variety of fruits, vegetables, and nuts (44), including soybeans (45). However, other studies have reported only marginal survival of plant-derived miRNAs under *in vitro* digestion conditions (46). The stability therefore appears to depend strongly on the food matrix, the miRNA species examined, and the digestion protocol applied, possibly explaining the apparent discrepancy. As previously stated, plant miRNAs in fact have shown overall higher stability due to their 3′ end 2′-O-methylation, which prevents uridylation and truncation (17), in addition to the protection conferred by the EVs in which several miRNAs have been found encapsulated (36). It is worth mentioning, though, that certain miRNAs such as miR-156e, miR-159, and miR-162 have been detected in feces following an acute plant-based diet (44, 46) but have not been found in serum (44). Interestingly, while current research has successfully quantified miRNAs in human digestive waste (44, 46), functional characterization remains limited by the natural yet complex biomolecular matrix of these samples. Specifically, the high abundance of cellular debris, dietary residues, and diverse microbial populations creates significant biochemical noise. This heterogeneity challenges the isolation process, making it difficult to discern whether extracellular miRNAs exist in a free form, are encapsulated within exosomes, or are stabilized by other mechanisms. Despite this, the miRNA detection outcomes were attributed to the amount of plant-derived food intake and the sensitivity of the methodologies employed, rather than a categorical absence of absorption. In order to determine if meaningful absorption and distribution of miRNA to peripheral organs can actually occur, closer scrutiny and further validation studies were warranted. Interestingly, a cross-sectional clinical study examined the expression of miR-168, a plant-derived microRNA from rice (*Oryza sativa*), in gastric tissues from patients with normal mucosa, chronic non-atrophic gastritis, atrophic gastritis, intestinal metaplasia, non-tumorous gastric tissue, and gastric cancer. This miRNA was consistently detected across all samples, showing higher levels in non-tumoral gastric mucosa than in tumoral tissue and greater expression in the stomach than in the colon. Its abundance was also higher in cases of moderate-to-severe atrophy and intestinal metaplasia (47).

The detection of plant-derived miRNAs across the hosts’ gastrointestinal tracts, waste products (44, 46), and even tissues (47) has led scientists to elucidate the protection mechanisms by which miRNAs are able to remain intact enough to be measurable. For this, RNA extraction processes have not only focused on miRNA sequencing, but also on physical characterization. As previously mentioned, the most satisfactory mechanism by which exogenous miRNAs are able to withstand degradation is through their natural encapsulation inside extracellular vesicles. Some of the comestibles that have revealed vesicular entrapment of miRNAs are apple (*Malus domestica*) (48), broccoli (*Brassica oleracea* var. *italica*) (49), ginger (*Zingiber officinale*) (50, 51), watermelon (*Citrullus lanatus*) (52), *Camellia sinensis* (53), garlic (*Allium sativum*) (54, 55), and *Centella asiatica* (56). The food items are usually ground so that their juice can be extracted and the EVs isolated, commonly by high-speed differential ultracentrifugation; however, some protocols might slightly differ.

Following the experimental workflow, free and EV-encapsulated miRNAs are purified, sequenced, and aligned to the host genome to identify putative targets, predict possible cross-kingdom miRNA-gene interactions, and assess further modulation. For instance, an extensive computational analysis of miR-167, miR-166, and miR-156, some of the most consistently reported miRNAs found in broccoli, exhibited close interaction with cellular proliferation mechanisms. Particularly, these miRNAs were correlated with ErbB and RAS signaling pathways, as well as with Protein Phosphatase 1 Regulatory Subunit 12B (PPP1R12B), a component of the myosin phosphatase complex, and V-Raf Murine Sarcoma Viral Oncogene Homolog B (BRAF), a protein involved in mitogenic signaling transduction (46). Meanwhile, other computational evaluations performed on different edible fruits, vegetables, and nuts identified miR-159, miR-162, and miR-156e in association with inflammatory pathways. In particular, miR-159 was associated with the negative regulation of insulin signaling and fatty acid β-oxidation, involving genes such as Provirus-Integrating Moloney virus site 3 (*PIM3*) and Insulin Receptor Substrate 1 (*IRS1*). Meanwhile, miR-162 was related to cytokine production and fatty-acid oxidation via Mitogen-activated Protein Kinase 14 (*MAPK14*); and the putative targets of miR-156e were enriched for the regulation of membrane permeability, bicellular tight junction assembly, and memory T-cell extravasation through F11 Receptor (*F11R*). Correspondingly, it was found that these three microRNAs had theoretical affinity with some bacterial genes, including medium-chain acyl-CoA synthetase (*FadK*), Chromosome Partition protein MukB, **S**erine/threonine-protein kinase B (*PknB*), sulfite reductase hemoprotein β-component (*CysI*), and exopolyphosphatase (*Ppx*) (44). While the documented in silico predictions might have offered a preliminary and promising framework, it remained necessary to establish whether the extracted EVs exerted biological activity, presumably through the effect of the miRNA cargo. In this manner, some researchers have evaluated the effects of plant-extracted miRNA-loaded EVs in human cellular models. The majority of these studies have been performed on colorectal adenocarcinoma (Caco-2) cells (50, 52), a gold-standard model of the human intestinal barrier that allows clear visualization of how molecules pass through the gut wall. One functional validation project that applied EVs isolated from watermelon juice (WMEVs) to this cell model revealed that this treatment altered the protein profile associated with apoptosis, cell movement, microtubule dynamics, lipid synthesis, and molecular transport. Indeed, previous RNA profiling revealed that miRNAs naturally present in WMEVs played roles in cell death, development, cytoskeletal motility, and proliferation. Since these activities have been related to placental development, EVs secreted by Caco-2 cells exposed to WMEVs were added to BeWo cells. Fluorescence labeling revealed that they are internalized by the placental trophoblast cell line BeWo via clathrin-mediated endocytosis, inducing proteomic changes that favored trophoblast differentiation along syncytial and invasive pathways, both essential for expanding the placental surface and enhancing nutrient transfer to the fetus. Taken together, these findings indicate that WMEVs are actively internalized by intestinal cells, thereby modifying the basal secretome and constituting a representation of human circulation after absorption, which in turn favorably altered placental function. Although the specific miRNAs and molecular targets responsible for these effects were not identified, this study provided a preliminary rationale for the effects of dietary WMEVs on fetal growth, gut-to-placenta communication, and pregnancy outcomes (52).

Other analyses with narrower specialization evidenced that miR-396 g-5p, miR-164 h-5p, miR-396a/b/f/g-5p, miR-164a/d/e, miR-2916, among other upregulated miRNAs found in ginger-derived exosome-like nanoparticles (GELNs) delivered into lipopolysaccharide (LPS)-stimulated Caco-2 cells had effects in inflammatory pathways, as they decreased the transcript levels of Nuclear Factor Kappa B (NF-κB), IL-6, IL-8, and TNF-α. Further, confocal microscopy showed that GELNs were internalized by these cells via caveolin-mediated endocytosis (51). Similar results were observed when the same treatment was applied to LPS-treated RAW264.7 cells, as the GELNs favored a shift from M1 toward the anti-inflammatory M2 phenotype, reducing the secretion of the pro-inflammatory cytokines TNF-α, IL-1β, and IL-6 and overexpressing the anti-inflammatory cytokine IL-10 (50). These results were consistent with the target-prediction results, as they revealed association with cellular and metabolic processes, such as cancer-related pathways, endocytosis, focal adhesion, Ras signaling, and TNF signaling cascades (51). Particularly, bioinformatic analyses exhibited that transforming growth factor-β-activated kinase 1-binding protein 1 (*Tab1*) was a target gene for miR-164d, and later, luciferase reporter assay verified their interaction, with NF-κB acting as a downstream effector (50). It was, of course, assessed if RNA-depleted GELNs had any significant effect, only showing a certain inhibitory effect on TNF-α (50). These findings suggest that natural miRNA-loaded GELNs confer protective effects against LPS-induced intestinal inflammation. The promising results, particularly those pointing in favor of miR-164d, led a group of scientists to synthesize miR-164d-enriched GELNs using a microfluidic device and a varied lipid cocktail for administration to DSS-induced colitis murine models (C57BL/6) via gavage. Correspondingly, the results included reduced inflammatory cell infiltration in the lamina propria. Moreover, colonic macrophages exhibited controlled expression of CD11b, reduced expression of IL-1β and IL-6, and elevated IL-10 and TGF-β, illustrating the potential of ginger-derived miR-164d in the control of intestinal inflammation (50).

EV-derived miRNAs from other edible plants were evaluated to assess their potential effects on inflammatory processes. One notable discovery was miR-3630-5p, highly abundant in garlic-derived exosome-like nanovesicles (GENs), which when exposed to Caco-2 cells stimulated with LPS displayed cytoplasmic accumulation and downregulation of Toll-like receptor 4 (TLR4), with consequent inhibition of myeloid differentiation primary response 88 (MyD88) and NF-κB. Functionally, its activity led to reduced secretion of pro-inflammatory cytokines IL-6, IL-1β, IFN-γ, and nitric oxide (NO), together with restoration of the tight junction proteins zonula occludens-1 (ZO-1), an adhesion protein, claudin-1, a sealing protein, and occludin, a barrier protein, thereby limiting epithelial damage and reestablishing the intestinal barrier’s integrity. Similarly, tea extracellular vesicles (TEVs) obtained from fresh juice of leaves from *Camellia sinensis* had immunomodulatory effects in LPS-treated macrophages (RAW264.7). Concretely, M1-associated mRNA Cluster of Differentiation 86 (*CD86*) and Macrophage Inducible Ca2^+^-dependent Lectin (*MINCLE*) were decreased, while the expression of M2 markers Cluster of Differentiation 206 (CD206) and Arginase-1 (ARG1) was elevated. Also, expression levels of TNF-α, IL-6, IL-1β, and inducible nitric oxide synthase (iNOS) were markedly reduced, while the level of IL-10 was augmented. Additionally, flow cytometry evidenced M2 polarization. Comparable effects were also observed in LPS-treated IEC-6 cells subjected to the same TEVs. These effects were predicted to be caused by miR-6300, miR-396a-3p and miR-166d-5p, contained in great abundance in the extracted *Camellia sinensis* extracellular vesicles. Notwithstanding, these cell-based findings must be cautiously interpreted. In both the ginger and tea models, intact vesicles were applied rather than purified, isolated miRNA species. In this sense, the observed anti-inflammatory phenotypes cannot be unambiguously and exclusively attributed to the miRNA cargo, as plant-derived vesicles commonly carry other molecules with recognized individual bioactivity (15, 35, 37). Moreover, the vesicle concentrations employed in these assays exceed those plausibly attainable by normal dietary intake, thus establishing biological plausibility rather than a demonstrated dietary effect. Therefore, in the absence of miRNA-specific approaches, such as miRNA depletion, mimic/inhibitor experiments, loss-of-function or rescue strategies, or direct target validation, the observed effects should be interpreted as activity of the intact EV preparation rather than as effects attributable specifically to individual miRNAs.

While administration of plant-derived EVs reported therapeutic efficacy in cellular culture paradigms, the precise molecular roles of the miRNA cargo still required mechanistic validation. Approaches including mimic transfections (45, 57) and application of enriched fractions (49) were mostly employed, demonstrating more evident biological effects. Namely, enriched fractions of miR-159a/b-3p, miR-403-3p, miR-219a, and miR-166b-3p initially obtained from broccoli were loaded into EVs via lipofection, achieving a 600-fold increase in relative expression levels. Interestingly, these engineered vesicles displayed elevated cytotoxicity even at low concentrations. In detail, Caco-2 cells exposed to a 5% vesicle fraction showed survival rates of approximately 30%. In contrast, naturally occurring EVs produced no discernible cytotoxic effect. The authors concluded that although no mRNA targets were evaluated in this approach, the ability of broccoli EVs to facilitate miRNA uptake while preserving miRNA stability underscores their potential as effective nanocarriers (49). Another experiment where differentiated human monocytic leukemia (THP-1) cells were cultured with miR-482f and miR-482c-5p mimics—identified across a variety of fruits, vegetables, legumes, and grains—evidenced that both plant-derived miRNAs could interact with these immune system cells, leading to the downregulation of pro-inflammatory genes such as ITAM Motif 1 (*NFAM1*), C-type Lectin Domain Family 7 Member A (*CLEC7A*), Tumor Necrosis Factor *(TNF),* and Interleukin 1 Beta (*IL1β*), coinciding with the computational prediction analysis. Additionally, miR-482f upregulated the expression of IL-10, an effect not observed for miR-482c-5p, which instead decreased THP-1-like macrophage viability by 13.5% (57). More importantly, other evaluations directly unveiled the miRNA-mRNA interactions that caused the biological mirroring. Special attention was drawn to miR-159a, highly concentrated in cooked and untreated soybean. Its mimic was transfected into Caco-2 cells, translating into a dose-dependent inhibition of colony formation alongside suppressed migratory capacity, and subsequent dual-luciferase tests confirmed the interaction of miR-159a and Transcription Factor 7 (*TCF7*) previously software-predicted, together with downstream inhibition of the proto-oncogene *MYC*, both of which are critical drivers of colorectal tumorigenesis via the Wnt/β-catenin signaling pathway (45). In parallel, another group of researchers co-transfected Human Embryonic Kidney HEK293 cells with a reporter plasmid containing the solute carrier organic anion transporter family member 2B1 gene (*SLCO2B1*) sequence and subsequently treated with apple-derived nanoparticles (APNPs). Luciferase activity declined after exposure to APNPs and was substantially reversed by inhibitors of miR-160a-e, −7121a-c, and -7121d-h, suggesting that multiple miRNAs present in the APNPs were responsible for modulating the intestinal transporter organic anion transporting polypeptide 2B1 (OATP2B1), a protein encoded by *SLCO2B1*. It was further detected that these three miRNAs formed a functional complex with immunoprecipitated AGO2 from APNP-exposed Caco-2 cells, corroborating their interaction with *SLCO2B1*. However, when mutated *SLCO2B1* constructs were in contact with APNPs, luciferase activity remained reduced, indicating a possible regulation beyond the studied miRNAs. Still, miR-160a-e, −7121a-c, and most prominently, −7121d-h did decrease the endogenous expression of OATP2B1 (48). Altogether, these findings revealed that interaction between dietary-derived miRNAs entrapped within EVs is possible, and that their effects are more pronounced when enriched fractions are artificially delivered.

To further elucidate if plant-derived miRNAs might have a significant effect not only in cells but in fully developed organisms, their delivery was performed mainly in C57BL/6 mice. For instance, to confirm that miR-159a had antitumorigenic effects as previously shown in Caco-2 cells, C57BL/6 mice with Azoxymethane (AOM)/Dextran Sulfate Sodium (DSS)-induced colorectal carcinogenesis received synthetic miR-159a via gavage. This group exhibited controlled total body weight, reduced spleen weight, and attenuated splenomegaly symptoms. Moreover, tumor incidence was considerably reduced, colon length was improved, and the number of Integrin alpha M (CD11b)-positive cells was reduced, indicating that this miRNA prevented the infiltration of immune cells. Furthermore, plasma levels of Interleukin-1 beta (IL-1β), Tumor Necrosis Factor alpha (TNF-α), Proliferating Cell Nuclear Antigen (PCNA), and Caspase-3 were correspondingly lower than in untreated cancer models (45). Nonetheless, most of the evidence from murine models currently focuses on intestinal anti-inflammatory effects. As the first evidence of the impact of raw EVs had in the C57BL/6 J murine model under DSS stimulation to induce inflammatory bowel disease (IBD), oral administration of GENs (100 mg kg^−1^) resulted in preservation of epithelial structure, restoration of tight junction proteins, and reduced inflammation (55). Interestingly, biodistribution studies revealed a predominant accumulation in the gastrointestinal tract, with minimal signal in liver, spleen, kidneys, and abdominal fat. GENs administered in DSS-induced acute colitis mouse models ameliorated symptoms, including intestinal bleeding and diarrhea. At the same time, serum pro-inflammatory cytokines IL-6 and TNF-α were correspondingly reduced, while ZO-1, Occludin, and Mucin 2 (MUC2) were preserved. Physiological evidence later confirmed mitigation of colon lesions (54). Similarly, *Centella asiatica*-derived exosomes (CAEs) administered orally to C57BL/6 J mice with induced acute ulcerative colitis showed selective accumulation at sites of colonic inflammation. Specifically, they exhibited colon elongation, increased body weight, a reduced disease activity index, and decreased myeloperoxidase activity, reflecting alleviation of inflammation, edema, and mucosal injury, all of which were later confirmed by histological analyses. Additionally, IL-6, IFN-γ, TNF-α, and IL-1β were markedly reduced, along with a higher level of IL-10, confirming the CAEs’ potential immunomodulatory activity. Subsequent single-cell sequencing analyses revealed that some of the CAEs-carried miRNAs could suppress the expression of Pseudopodium-Enriched Atypical Kinase 1 (*PEAK1*) and predicted binding affinities with Gephyrin (*GPHN*), leucyl-tRNA synthetase 2 (*LARS2*), and Ribosomal Protein L36 (*RPL36*) (56). It is encouraging to note that similar effects were also reported in Specific Pathogen Free Bagg Albino Laboratory-Bred Strain c (BALB/c). For example, TEVs administered specifically to this murine strain with DSS-induced colitis alleviated colon shortening and body weight loss. Histological examination evidenced recovery of ulceration and barrier breakdown. Molecular mechanisms were examined via dual-luciferase reporter assays, revealing that miR-166d-5p and miR-396a-3p, contained within the TEVs, specifically bind to mammalian target genes Inhibitor of Nuclear Factor Kappa B Kinase Subunit Beta (*IKBKB*) and AKT serine/threonine kinase 1 (*AKT1*), thereby regulating macrophage polarization through inhibition of NF-κB (53). To further evaluate the effect of a single plant-derived miRNA, chemically synthesized miR-159, abundant in broccoli, was gavage-fed, and after 7 days, hematoxylin-and-eosin staining revealed clearly defined and regularly arranged intestinal villi. Meanwhile, ELISA assay did not show significant increases in IL-2, TNF-α, Interferon-gamma (IFN-γ), or IL-6 (58). These findings, taken together, therefore provided evidence that, at least for the studied plant-derived miRNAs, when orally administered, they possess local anti-inflammatory effects. However, it should be emphasized that these outcomes were obtained under controlled conditions, mainly through gavage administration of synthetic/purified miRNA or artificially enriched vesicles, rather than through normal dietary consumption. Moreover, the absence of a significant cytokine increase might indicate the lack of a detectable pro-inflammatory response rather than constituting a definitive anti-inflammatory effect; whether comparable physiological outcomes are achievable through usual dietary doses remains an open debate. Accordingly, the reviewed studies can be distinguished according to whether miRNAs were delivered through normal dietary consumption, naturally occurring food-derived EVs, purified or synthetic miRNAs/mimics, or enriched/engineered EV preparations.

Given the growing evidence that plant-derived miRNAs affect intestinal inflammation and other gut-related illnesses, their possible correlation with the naturally occurring microbiota was inherent and therefore explored using diverse approaches. It was seen that the previous GENs that had already provided evidence for anti-inflammation properties in Caco-2 cells and C57BL/6 J mice modulated the intestinal microbiota by increasing the population of the *Lachnospiraceae* family, while decreasing the relative abundance of pro-inflammatory genera such as *Helicobacter* and *Escherichia–Shigella* when evaluating the feces of mice that had previously been gavage-fed with GENs (55). To further explore their impact on intestinal bacteria, a separate study cocultured GENs with gut microbiota isolated from mouse feces. Fluorescence microscopy revealed bacterial uptake of the nanoparticles. Then, diversity analyses revealed that gut microbiota richness increased after EV intervention, and that the Firmicutes-to-Bacteroidetes (F/B) ratio was restored. Particularly, the relative abundance of *Eubacterium ruminantium*, *Insolitispirillum*, *Megasphaera*, and *Acetatifactor* was elevated, while that of *Anaerostipes*, *Anaeroplasma*, *Ileibacterium*, and *Dubosiella* declined. More interestingly, BLAST alignment indicated that miR-2916-p3/p5 had putative binding sites with *Bacteroides thetaiotaomicron,* a highly abundant commensal bacterium essential for intestinal homeostasis and colon health. Coculture of this species and both miRNAs revealed that miR-2916-p3 rather than miR-2916-p5 significantly stimulated the growth of this bacterium (54). Meanwhile, a more detailed analysis of the intestinal samples from broccoli-derived miR159-fed BALB/c mice showed significant increases in *Weissella*, *Bacteroides*, *Ralstonia*, *Blautia*, and *Bifidobacterium* at the genus level, and in Proteobacteria at the phylum level. In addition, it was observed that the F/B ratio was significantly lowered compared to that in the negative control groups, which suggested miR-159’s role in maintaining normal intestinal homeostasis. *In vitro* bacteria-miRNA co-culture assays confirmed that intact miR-159 presented inhibitory effects on *Bacillus* sp. while promoting growth on *R. eutropha* and *W. paramesenteroides*. These bacteria were then analyzed using fluorescence microscopy and flow cytometry, confirming the internalization of intact, CY3-labeled miR-159. According to a dual-luciferase reporter assay performed in external bacterial cultures, miR-159 potentially targeted the bacterial genes cellulase C (*CelC*), RNase Y (*RnY*), and polyhydroxyalkanoate depolymerase 2 (*PhaZ2*), which may represent the mechanism by which miR-159 modulates the gut microbiota (58). On the other hand, other publications exhibiting a less-specific level of evidence indicated that CAEs that were orally administered to C57BL/6 J mice with induced acute ulcerative colitis provoked a reduction of pathogenic species *Salmonella enterica*, *Klebsiella pneumoniae*, and *Streptococcus oralis*, while increasing the relative abundance of beneficial species *Blautia hansenii*, *Pseudoflavonifractor capillosus*, and *Alloprevotella rava*. While CAEs exhibited a degree of capacity to modulate immune responses and regulate the gut microbiota through their natural miRNA cargo, the association of their target genes with ulcerative colitis requires deeper experimental evidence (56).

After a thorough literature search, to the best of our knowledge, there is no currently reliable or extensive published research on human trials addressing the molecular or physiological effects of orally administered plant-derived miRNAs. In fact, only an integrative analysis where the expression of miR-168, a plant-derived microRNA from rice was examined in the stomach tissues from patients with different types of gastritis, metaplasia, and cancerous tissue revealed that a trend toward improved prognosis and survival was noted among tumorous gastric cancer patients, as they exhibited higher concentrations of miR-168. Also, in the same study, significant associations between miR-168 levels and epigenetic markers, including the miR-137 promoter, DNA methylation, and Long Interspersed Nucleotide Element-1 (LINE-1), were reported. Although the mechanistic bases for these correlations and differences remain undetermined, it is speculated that the potential internalization of miR-168 might be more difficult in tumorous tissues. The evidence of this research remains associative; therefore, the leap toward demonstrated causal function is still pending (47).

### 2.2 Animal-derived miRNAs directing cross-species communication

As previously mentioned, edible plants are not the only sources of dietary exogenous miRNAs, as milk is also a relevant source of these species and might mediate a molecular-based cross-species communication. To address this possibility, total RNA was extracted from cow and yak milk exosomes and sequenced. Results revealed that miR-31, miR-34a, miR-10b, miR-29c, miR-2284X, and miR-186 were upregulated, while miR-6524, miR-183, miR-182, miR-215, miR-30d, and miR-let-7b were downregulated in yak relative to cow milk. KEGG pathway analyses linked these miRNAs to hypoxia-related proteins. In particular, miR-34a stood out as a candidate regulator of genes involved in the vascular endothelial growth factor signaling pathway, including Vascular Endothelial Growth Factor A (*VEGFA*), Caspase 9 (*CASP9*), and Mitogen-Activated Protein Kinase 13 and 14 (*MAPK13/14*), among others. To further evaluate its biological impact, miR-34a mimic was transfected into intestinal epithelial cells (IEC-6). The MTT assay indicated that the survival rate of the cells under hypoxic conditions increased by 13 and 9.5% compared with the negative control group at 12 and 24 h post-transfection, respectively. Mechanistically, miR-34a promoted the expression of prolyl hydroxylase-1 (*PHD-1*) while decreasing the expression of Hypoxia-Inducible Factor alpha (*HIF-α*) and its downstream target Vascular Endothelial Growth Factor A (*VEGFA*), both of which are commonly overexpressed under hypoxic conditions. Collectively, these results indicate that miR-34a supports IEC-6 survival under controlled, isolated hypoxic stress, which may aid in preserving the epithelial barrier and restoring intestinal barrier function (59).

These milk-based studies converge substantially at the mechanistic level. For instance, both coincide in milk extracellular vesicles as the natural delivery vehicle for miRNAs, exhibiting comparable resistance to gastric conditions and similar endocytic routes for intestinal internalization. However, these similarities should not be interpreted as evidence that consumption of either milk directly promotes intestinal health, mainly because the contribution of individual miRNAs cannot necessarily be distinguished from that of other vesicular components. Nevertheless, these studies broaden the scope of dietary exogenous miRNA research beyond plant-derived sources and suggest that animal-derived milk vesicles may also provide a biologically relevant vehicle for miRNA delivery to the gastrointestinal tract.

### 2.3 Maternal-milk miRNAs executing intraspecies crosstalk

Although miRNAs from animal and human milk may share similar routes of oral delivery and gastrointestinal interaction, these contexts are distinguished according to the biological relationship between the miRNA source and recipient. MiRNAs derived from animal milk consumed by humans are considered in the context of cross-species communication, in which dietary miRNAs originating from one species may interact with biological systems of another species. In contrast, maternal-milk miRNAs are discussed as part of maternal-offspring intraspecies crosstalk, reflecting the potential vertical transfer of molecular signals. Despite this distinction, both contexts may involve common processes related to the protection of miRNAs within milk or extracellular vesicles, their stability during gastrointestinal transit, and their potential interaction with intestinal cells and local immune components. However, the extent to which the processes result in biologically relevant effects may depend on the specific miRNA, its abundance and stability, as well as the physiological context of the recipient. Thus, the distinction between these two sections relies primarily on the communication rather than on fundamentally different mechanisms of gastrointestinal stability or uptake.

A comprehensive study, which included *in vitro*, *in vivo*, and direct evidence of molecular interactions, focused on the miRNA content of raw milk from sows (*Sus scrofa domesticus*) supplemented with resistant starch. It was found that, within the milk extracellular vesicles, miR-6516, miR-1285, miR-223, miR-9841-3p, miR-18a, and miR-146a-5p were significantly upregulated. When analyzed separately, the miR-146a-5p mimic most effectively improved cell viability in the Intestinal Porcine Epithelial Cell line (IPEC-J2). Equally important, intact milk extracellular vesicles increased wound healing rates from 10 to 18% when incubated with this same cell line. Predictive analyses revealed neural precursor cell-expressed developmentally downregulated 4-like (*NEDD4L*) as a putative target gene of miR-146a-5p, which was later validated by luciferase assays. Further *in vitro* analyses showed that transfection of IPEC-J2 cells with miR-146a-5p mimics increased the levels of Disheveled Segment Polarity Protein 2 (DVL2) and β-catenin, thereby activating the intestinal Wnt pathway. *In vivo* oral administration of these vesicles to C57BL/6 mice triggered body weight gain, longer intestine, increased villus length, augmented jejunal crypt depth, upregulated Cyclin D1 (CCND-1), and cellular proliferation; effects mirrored upon injection of agomiR-146a-5p. In general, this body of evidence indicated that miRNAs present in porcine milk extracellular vesicles might influence the intestinal development of their offspring (60). While sow milk is not ordinarily consumed, this study was included as it encompasses *in vitro*, *in vivo*, and reported, validated evidence. In addition, the porcine gastrointestinal tract is considered a relevant translational model for studying aspects of human intestinal physiology.

It was reported that in women less than 15 days postpartum who had followed a plant-based diet, 30 miRNAs were downregulated, while only miR-143-3p was upregulated. Computational analyses associated the downregulated species with Toll-like receptors TLR-3, −4, −5, −7, −8, −9, and −10, as well as Interleukin 17 (IL-17), Myeloid Differentiation Primary Response 88 (MyD88), and the Phosphoinositide 3-kinase/ Protein Kinase B (PI3K/AKT) axis. Conversely, miR-6783-5p, miR-658, miR-6511a-5p, miR-3125, miR-6747-5p, and miR-5093 were abundant in the cluster of women under an animal protein diet. Significant associations between these miRNAs and the microbiota of infants who consumed that milk were identified, regardless of the mother’s diet. At the phylum level, positive correlations emerged with Firmicutes, Actinobacteria, and Proteobacteria: taxa central to early gut colonization and to metabolic and immune development. Meanwhile, at the genus level, *Ruminococcus, Serratia, Micrococcus, Finegoldia,* and *Peptoniphilus* were positively associated. Interestingly, Blautia, Gemmiger, and Fusicatenibacter were tightly connected with miR-195, miR335 (reported as “miR-195-bp” and “miR335-bp,” respectively, in the original study; the corresponding 5p/3p strand designation was not specified), miR16-5p, miR26b-5p and miR-30c-5p; whereas miR-6802-5p, miR-2467-3p, miR-4441, miR-3937, miR-6778-5p, miR-4451, miR-2392, miR-128-1-5p, miR-4269, and miR-1238-5p were closely linked to Veillonella and Flavonifractor. Pathway enrichment analysis revealed interactions with signal transduction, tyrosine-kinase signaling, Rho GTPase activity, and membrane trafficking, suggesting that maternal milk miRNAs have a role in the early shaping of the consumer’s gut microbiota and other broader host signaling mechanisms; however, these associations remain correlational, thus causal validation through functional assays would provide more evidence of the underlying causes (61). A concise summary of information on the miRNA source, experimental exposure, models used, biological effect, evidence strength, and limitations of the cited studies is provided in Table 1.

**Table 1:** Summary of the current evidence of dietary exogenous miRNAs and their effect on cells, murine models, tissues, bacteria, and human cohorts, including the evidence strength and limitations for each.

miRNA source	Experimental exposure	Model(s)	Direct target evidence	Biological effect	Evidence strength	Major limitation	References
Broccoli (miR167 ↑, miR166 ↑, miR156 ↑, miR165a-5p ↑, miR168a ↑, miR167a/b/c ↑, miR165b ↑, miR166a/b/c/d/e ↑)	*In vitro* simulated digestion.	*In silico*. Simulated *in vitro* digestion.	–	Predicted interaction with ErbB/RAS pathways. Marginal post-digestion survival.	Computational.	No cellular or functional validation of predicted targets.	(46)
Various raw/cooked fruits, vegetables, and nuts (miR156e ↑, miR159 ↑, miR162 ↑)	Three-day natural acute dietary intake in humans.	*In silico* prediction and human feces/serum.	–	Detected in all fecal samples, absent in serum. Predicted insulin signaling (negative regulation) and fatty-acid β-oxidation (miR159); Cytokine production and fatty-acid oxidation (miR162); membrane permeability, tight-junction assembly, T-cell extravasation (miR156e).	Computational prediction and correlational approach.	No functional validation of predicted targets and systemic absorption unconfirmed.	(44)
Human milk (miR-143-3p ↑; miR-6783-5p ↑, miR-658 ↑, miR-6511a-5p ↑, miR-3125 ↑, miR-6747-5p ↑, miR-5093 ↑, among others)	Breastfeeding.	*In silico*: infant microbiota correlation.	–	Correlational associations with signal transduction; tyrosine-kinase signaling; Rho GTPase activity; membrane trafficking, PI3K/AKT, Firmicutes, Actinobacteria, Proteobacteria, *Ruminococcus, Serratia, Micrococcus, Finegoldia,* and *Peptoniphilus*	Computational prediction and correlational approach.	No functional tests were performed. Effects can be attributed to other milk components.	(61)
Broccoli (miR159a/b-3p ↑, miR403-3p ↑, miR219a ↑, miR166b-3p ↑)	Cell direct addition of enriched vesicles.	Caco-2 cells.	–	Efficient delivery and uptake of exogenous miRNAs by Caco-2 cells; miRNA-loaded engineered broccoli EVs markedly reduced cell viability, whereas naturally occurring EVs showed no comparable cytotoxicity.	*In vitro* delivery/viability evidence; not direct target-specific validation.	No functional analyses were performed.	(49)
Rice (miR-168 ↑)	Natural dietary intake.	Human gastric tissue biopsies.	–	Candidate biomarker in gastric pathology and regulator of intestinal homeostasis; associated with methylation; trend toward improved prognosis of patients with tumorous gastric cancer.	Correlational. No further validation tests were performed.	Mechanism by which the miRNA was present in the tissues was undetermined.	(47)
Yak/cow milk exosomes (miR-34a ↑, miR-31 ↑, miR-10b ↑, miR-29c ↑, miR-2284X ↑, miR-186 ↑; miR-6524 ↓, miR-183 ↓, miR-182 ↓, miR-215 ↓, miR-30d ↓, miR-let-7b ↓)	miR-34a mimic transfected to intestinal epithelial cells.	IEC-6 cells.	Protein detection via Western-blot.	Survival under hypoxia (+13%/+9.5% at 12 h/24 h); PHD-1↑, HIF-α↓, VEGFA↓	Target protein expression.	Synthetic mimic without *in vivo* validation nor studied physiological effects.	(59)
Apple nanoparticles (miR-160a-e ↑, miR-7121a-c ↑, miR-7121d-h ↑)	Purified nanoparticle exposure + synthetic mimic/inhibitor.	Caco-2 and HEK293 cells.	Luciferase reporter and AGO2 co-immunoprecipitation.	*SLCO2B1/OATP2B1 ↓*	Mechanistically validated *in vitro* through fluorescence expression.	No *in vivo* validation.	(48)
Soybean (miR159a ↑)	Synthetic mimic in cells + gavage feeding to mice.	Caco-2 cells and AOM/DSS-induced colorectal carcinogenesis mice (C57BL/6)	Dual-luciferase.	↓ colony formation/migration in Caco-2; ↓ tumor incidence, ↓ immune infiltration, ↑ body weight, ↓ spleen weight, ↓ splenomegaly in DSS colorectal carcinogenic mice	*In vitro*, *in vivo*, and fluorescence reporter-validated.	Non-physiological gavage dose.	(45)
Ginger (miR396g-5p, miR164h-5p, miR396a/b/f/g-5p, miR164a/d/e, miR2916)	EV administration to cells.	LPS-stimulated Caco-2 cells.	–	Protective against LPS-induced inflammation in Caco-2 cells.	*In vitro*, not miRNA-specific.	No direct target validation and no single-miRNA analysis. Effects might be attributed to other EV components.	(51)
Ginger (miR164d ↑)	Synthetic miR164d-enriched EVs gavage-fed and natural EVs tested *in vitro*.	RAW264.7 macrophages and DSS-colitis mice (C57BL/6)	Luciferase-confirmed Tab1 target	M1 → M2 macrophage shift; ↓TNF-α/IL-1β/IL-6, ↑IL-10; *in vivo* ↓ inflammatory cell infiltration, preserved epithelial architecture, controlled expression of CD11b, ↑TGF-β	*In vitro, in vivo,* and reporter-validated.	Not achievable with natural dietary exposure.	(50)
Watermelon (individual miRNAs not tested)	Purified EV administration applied *in vitro*.	Caco-2 → BeWo (intestinal –placental secretome model).	–	Altered Caco-2 apoptosis, motility, microtubule dynamics, lipid synthesis, transport; induced BeWo trophoblast differentiation	No miRNA-specific mechanism elucidated.	No individual miRNA identified or validated. Effects might be attributed to other EVs components	(52)
Variety of fruits, vegetables, legumes, and grains (miR482f, miR482c-5p)	Synthetic mimic transfection to cells and natural dietary intake in humans.	THP-1 macrophages and human cohort.	mRNA expression analysis via qPCR.	↓ macrophage viability; detected in feces, but not in serum. *NFAM1 ↓, CLEC7A ↓, TNF ↓, IL1β ↓, IL-10 ↑; TLR6 ↓*	Cellular mRNA expression and human detection.	Serum absorption unconfirmed in human cohort.	(57)
Sow milk (miR-6516 ↑, miR-1285 ↑, miR-223 ↑, miR-9841-3p ↑, miR-18a ↑, and miR-146a-5p ↑)	Purified EVs, synthetic miRNA mimic, and *in vivo* oral EV administration *Not commonly a part of the human diet. Included because of the strength of the evidence.	IPEC-J2 cells and C57BL/6 mice.	Luciferase-confirme d NEDD4L target.	↑ IPEC-J2 viability/wound healing; ↑ body weight, ↑ villus length/crypt depth, ↑CCND1, ↑ cellular proliferation *NEDD4L ↓; DVL2 ↑, β-catenin ↑, CCND1 ↑*	*In vitro, in vivo* and reporter-validated.	Not validated in humans.	(60)
*Camellia sinensis* (miR6300 ↑, miR166d-5p ↑, miR396a-3p ↑)	Varying concentrations of purified EVs	RAW264.7 and IEC-6 cells; DSS-colitis mice (BALB/c).	Dual luciferase confirmation: IKBKB-miR166d-5p and AKT1 -miR396a-3p.	M1 → M2 polarization; ↓TNF-α/IL-6/IL-1β/iNOS, ↑IL-10; ↓ colon shortening/weight loss *CD86 ↓, MINCLE ↓, CD206 ↑, ARG1 ↑, IKBKB ↓, AKT1 ↓, NF-κB ↓*	*In vitro, in vivo* and Reporter-validated with fluorescence expression.	Most abundant species (miR6300) not functionally tested. Causes might be also attributed to other components of the EVs.	(53)
Broccoli (miR159 ↑)	Synthetic miRNA oral administration to mice and direct bacterial coculture.	BALB/c mice and bacterial coculture.	Dual-luciferase in bacterial culture confirmed bacterial targets.	Normal ileal histology, no cytokine increase; ↑ microbial diversity; ↓ F/B ratio; ↓ *Bacillus* sp. growth, ↑ *R. eutropha*/*W. paramesenteroides CelC ↓, RnY ↓, PhaZ2 ↓*	Direct bacterial-target validation and *in vivo* administration of the synthetic miRNA.	Synthetic miRNA gavage, not achieved via natural diet.	(58)
Garlic (miR3630-5p ↑)	Purified EV administration *in vitro* and *in vivo*.	LPS-stimulated Caco-2 cells and DSS-colitis mice (C57BL/6 J)	Protein expression evaluated with Western Blotting and dual luciferase verification.	↓ IL-6, IL-1β, IFN-γ, NO; restored ZO-1, claudin-1, and occludin; preserved epithelium, ↓ inflammation; ↑ Lachnospiraceae, ↓ *Helicobacter*/ *Escherichia–Shigella* TLR4 ↓, MyD88 ↓, NF-κB ↓	*In vitro, in vivo,* and verification of expression with luciferase and Western Blot.	Not evaluated in human cohort.	(55)
Garlic (miR2916-p3/p5 ↑)	Purified EV administered *in vivo* and direct bacterial coculture with isolated miRNA.	DSS-colitis mice (C57BL/6 J) and gut microbiota coculture + *B*. *thetaiotaomicron* coculture.	ELISA validation and immunofluorescence biodistribution confirmation.	Ameliorated DSS colitis, ↓bleeding/diarrhea, preserved ZO-1/occludin/MUC2; ↑ microbiota richness, restored F/B ratio; ↑ *Eubacterium ruminantium*, *Insolitispirillum*, *Megasphaera*, and *Acetatifactor*, ↓ *Anaerostipes*, *Anaeroplasma*, *Ileibacterium*, and *Dubosiella*; ↑ *B*. *thetaiotaomicron*	Direct bacterial mechanistic *in vitro* validation and *in vivo* assay.	Not validated with human cohort.	(54)
*Centella asiatica* (miR159, miR396c-3p, miR396b)	Purified EV with administration *in vitro* and *in vivo*.	RAW264.7/NCM460 cells, ulcerative colitis mice (C57BL/6 J) and single-cell sequencing.	Single-cell sequencing determined gene expression.	Colon elongation, ↑body weight, ↓disease activity index, ↓myeloperoxidase; ↓ *Salmonella enterica*, *Klebsiella pneumoniae*, and *Streptococcus oralis*, ↑ *Blautia hansenii*, *Pseudoflavonifractor capillosus*, and *Alloprevotella rava*	*In vivo* phenotypic and predictive target data via gene expression.	Microbiota growth was only evaluated with optical density. No functional validating analyses were performed.	(56)

An overview of the dietary exogenous miRNAs evaluated in the literature, their dietary sources, and the main reported outcomes across different evidence domains is presented in Figure 2. The available evidence spans detection and computational analyses, cellular models, *in vivo* studies, and gut microbiota-related findings. Detection and computational studies mainly provide evidence of miRNA presence, predicted targets, and associations with microbial composition, whereas cellular and *in vivo* studies report effects on inflammatory signaling, epithelial integrity, immune responses, and disease-associated phenotypes. Studies involving the gut microbiota additionally report changes in microbial diversity, bacterial growth, and potential miRNA–bacterial gene interactions. Together, these findings illustrate the diversity of experimental approaches and biological outcomes reported for dietary exogenous miRNAs, while also highlighting the heterogeneity of the current evidence.

**Figure 2:**
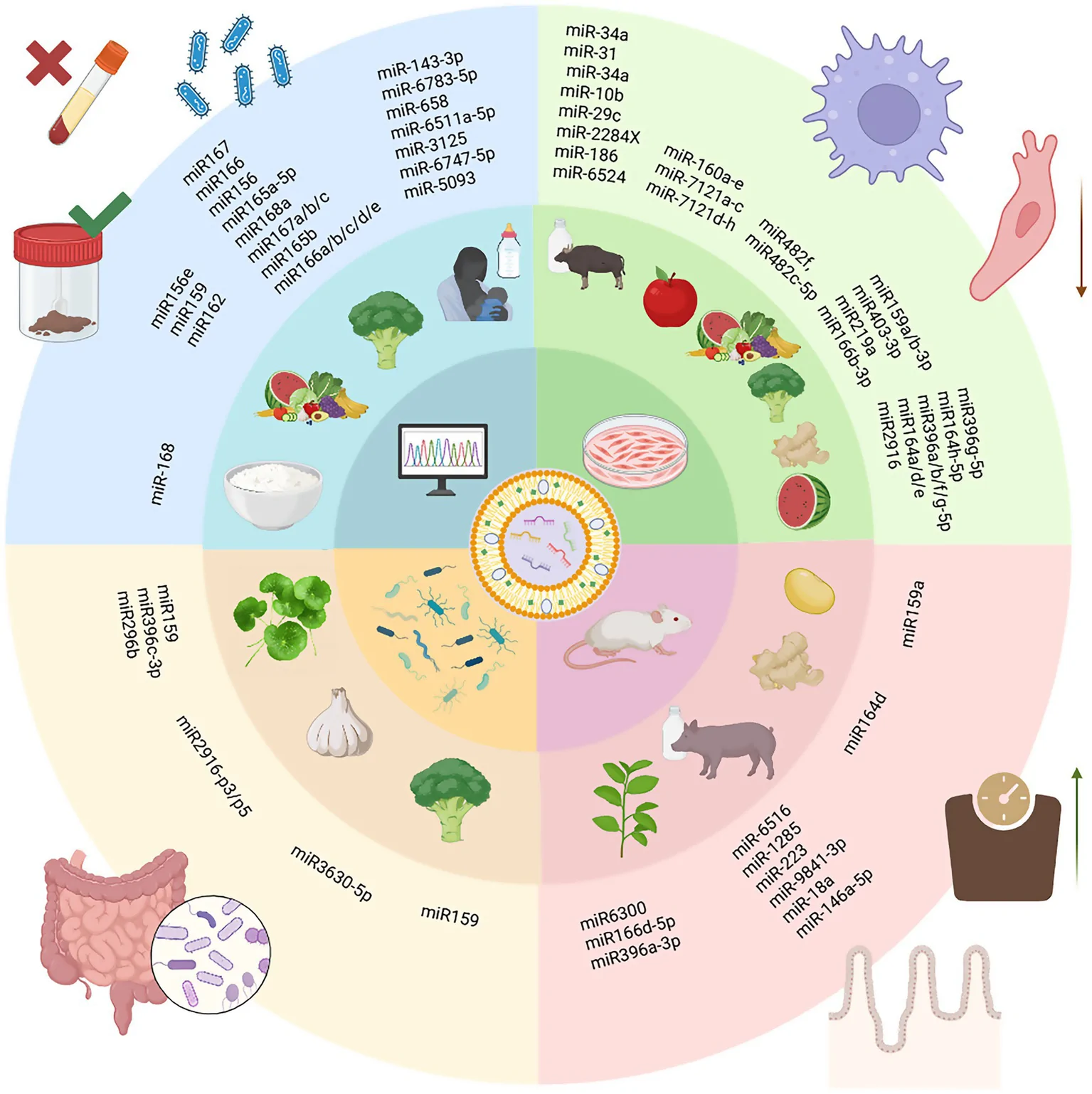
Schematic overview of the dietary exogenous miRNAs evaluated across detection and computational (blue), cellular (green), *in vivo* (pink), and gut microbiota (yellow) evidence domains, together with their dietary sources and representative reported outcomes. Detection and computational evidence include miRNA detection in feces, lack of consistent detection in serum, predicted host or microbial targets, and associations with microbiota composition. Cellular evidence includes modulation of inflammatory signaling, M2-like macrophage polarization, and reduced cellular migration. *In vivo* evidence includes preservation of intestinal architecture, attenuation of inflammatory responses, and changes in body weight or disease-associated phenotypes. Microbiota-related evidence includes changes in microbial diversity and composition, bacterial growth, and potential miRNA–bacterial gene interactions. Some miRNAs have been omitted from the figure for visual clarity. Image created in BioRender.com.

## 3 Shared biological functions of dietary exogenous miRNAs

The three miRNA sources discussed throughout this text share certain biological effects. This section aims to summarize the evidence by biological outcome rather than origin, providing a novel perspective and point for comparison.

### 3.1 Intestinal development and epithelial barrier integrity

The most recurrent effect of the dietary-derived exogenous miRNAs concerns the preservation, usually accompanied by restoration, of the intestinal epithelium. For instance, miR-34a mimic, found upregulated in yak milk, increased the survival rate of epithelial cells under hypoxic conditions, presumably by decreasing the expression of *HIF-α* and *VEGFA* while promoting *PHD-1*, a mechanism relevant to barrier maintenance under ischemic conditions (59). Sow milk-mimicked miR-146a-5p accelerated wound healing in IPEC-J2 and *in vivo*, increasing villus length, jejunal crypt depth, and CCND1-associated cellular proliferation, with validation via increased levels of DVL2, β-catenin, and further activation of the Wnt pathway (60). Among vegetal sources, GENs applied to Caco-2 cells displayed limited epithelial damage and restoration of the intestinal barrier integrity. Similar outcomes were seen when these EVs were delivered orally to mice, as the analyses revealed preservation of epithelial structure, restoration of tight junction proteins, and reduced bowel inflammation (55). It is notable that developmental and barrier-directed effects are where dietary-derived miRNAs provide the strongest mechanistic evidence.

### 3.2 Inflammatory signaling and immune cell responses

Modulation of inflammatory signaling has been reported predominantly for plant-derived miRNAs. Convergent findings across independent studies implicate the NF-κB pathway: GELNs applied to Caco-2 cells (51) and RAW264.7 cells through Tab1 (50); miR-166d-5p and miR-396a-3p from TEVs through specific binding to IKBKB and AKT1 (53); and GEN-derived miR-3630-5p through the TLR4/MyD88/NF-κB cascade (55). At the cellular level, ginger- and tea-derived EV preparations were reported to promote macrophage polarization toward an anti-inflammatory M2-like phenotype, accompanied by reductions in TNF-α, IL-1β, and IL-6 and increases in IL-10 (50, 53). Other plant-derived miRNAs or EV preparations also showed anti-inflammatory effects through different mechanisms (45, 51, 54, 56, 58). Moreover, plant-derived miR-482f and miR-482c-5p were associated with regulation of NFAM1, CLEC7A, TLR6, TNF, IL1β, and IL-10 (57). Emerging evidence suggests that EVs originating from certain probiotic bacteria may also carry miRNA-like small RNAs capable of modulating host inflammatory responses. In a recent study, orally administered *Lactobacillus reuteri*-derived EVs (LrEVs) were detected in the gastrointestinal tract of BALB/c mice. LrEVs contained abundant miR-4239, which was shown by dual-luciferase reporter assay to bind directly to the 3′-UTR of IL-17A. In H1N1-infected mice, LrEV treatment reduced intestinal TNF-α, IL-1β, and IL-6 while increasing IL-4, and partially ameliorated infection-associated alterations in the gut microbiota. Importantly, this evidence should be regarded as an emerging and adjacent mechanism rather than as directly equivalent to plant- or animal-derived dietary miRNA communication, because bacterial small RNAs are mechanistically distinct from canonical eukaryotic miRNAs and the intervention involved isolated probiotic-derived EVs rather than normal dietary consumption. Collectively, these independent findings support the potential of food- and probiotic-associated EV small RNAs to modulate inflammatory signaling through distinct molecular pathways; however, most evidence has been obtained using purified, synthetic, or experimentally administered preparations rather than through ordinary dietary exposure (62).

### 3.3 Cellular proliferation and tumor-associated pathways

A smaller set of studies addressed proliferative and tumorigenic pathways in the gastrointestinal tract. Soybean-derived miR-159a mimic suppressed colony formation and migration in Caco-2, while reducing tumor incidence in AOM/DSS-treated mice, validated via targeting of TCF7 and downstream *MYC* repression within the Wnt/β-catenin axis (45). Broccoli-derived EVs proved cytotoxic to Caco-2 cells only when artificially enriched, with predicted targets being associated with cancer transcriptional misregulation (49).

### 3.4 Modulation of intestinal microbial communities

Evidence that dietary miRNAs act upon the gut microbiota is relatively distinctive, since it implies an additional kingdom regulatory boundary. A chemical mimic of broccoli-derived miR-159 fed to mice exhibited a significant increase in the relative abundance of *Weissella*, *Bacteroides*, *Ralstonia*, *Blautia*, and *Bifidobacterium* at the genus level and Proteobacteria at the phylum level, with a lower F/B ratio, and targeting of bacterial genes *CelC*, *RnY*, and *PhaZ2* (58). Meanwhile, GENs administered orally to mice exhibited an increase in the population of the *Lachnospiraceae* family, while decreasing *Helicobacter* and *Escherichia–Shigella* (55); and, when evaluated *in vitro*, restoration of the F/B ratio and a particular abundance of *Bacteroides thetaiotaomicron* (54). Additionally, CAEs administered to mice showed a reduction of *Salmonella enterica*, *Klebsiella pneumoniae*, and *Streptococcus oralis*, while increasing the relative abundance of *Blautia hansenii*, *Pseudoflavonifractor capillosus*, and *Alloprevotella rava* (56). The microbiota shifts after whole EVs administration, however, cannot be distinguished from indirect consequences of reduced inflammation or barrier function restoration; therefore, it should not be interpreted as evidence of direct bacterial targeting. Maternal milk miRNAs have likewise been associated with changes in the infant microbiota composition, but the evidence remains observational (61).

## 4 Critical appraisal of mechanistic evidence, controversies, and translational potential

Although the mechanistic evidence compiled in this review provided proof-of-concept throughout diverse approaches that dietary exogenous miRNAs can modulate host gene expression as well as the composition of the intestinal microbiota under controlled experimental conditions (Figure 2), a recurrent pattern emerges upon comparing these findings with detection studies conducted in humans following a defined diet: fecal miRNA detection rarely translates into quantifiable serum levels. This discrepancy raises questions about whether the biological effect of dietary exogenous miRNAs is predominantly local, depends on limited systemic absorption, or is masked by current detection methods.

While the evidence is substantial at the computational and cellular levels, its extrapolation to whole living organisms is still questioned. Several bioinformatic databases recognize and integrate complementary sequences between dietary exogenous miRNAs and animal transcripts, converging in the fact that plant and animal genomes share sequence architecture consistent with canonical miRNA-mRNA target interactions. Although this constitutes a necessary first step toward exploring trans-kingdom miRNA transfer, experimentally reproducible *in vitro* and *in vivo* validation remains indispensable.

Among the most frequently invoked arguments against the possibility of cross-kingdom miRNA regulation are the persistent and contradictory findings regarding the stability, abundance, mechanisms of action, validation, and potential targets of plant-derived miRNAs in human cells and individual organisms (20). Several studies indicate that the dietary miRNA content is too low to elicit meaningful modulation of gene expression (63). This has been attributed by several authors to experimental artifacts, insufficient sample sizes, and inadequately optimized bioinformatic pipelines. In a large meta-analysis, 133 miRNAs drawn from seven independent studies were analyzed. The evaluators concluded that the transfer of exogenous dietary miRNAs into host tissues does not occur under all conditions. This outcome was supported by the markedly low expression of exogenous dietary miRNAs across samples, making them unlikely to be sufficient to regulate the hosts’ cellular transcripts. However, under certain conditions and periods, they conceded that exogenous dietary miRNAs can regulate immunity (14).

Other investigators counter-argue that absorption barriers may be overcome through the development of advanced, targeted oral delivery systems. For instance, synthetic nanocarriers, engineered plant exosomes, and microbial carriers are among the alternatives, as they offer biocompatibility, stability, barrier penetration, and tissue targetability. Nevertheless, scalability, long-term safety, purification complexity, and lack of standardization remain among the field’s most pressing challenges (64).

*In vitro* assays have consistently reflected benefits at the tissue level within the intestine, as reported by Gao et al. (59), Komori et al. (48), Liu et al. (45), Yin et al. (51), Yan et al. (50), Timms et al. (52), Luo et al. (53), and Shi et al. (56). Nonetheless, while these results might provide a proof-of-concept evidence for food-derived miRNA-mediated regulation under controlled experimental conditions, translating these findings to living organisms faces several major hurdles. For instance, *in vitro* assays, by design, are performed on a selected cluster of isolated cells, to which relatively high doses of purified miRNAs are added directly. Extrapolating this scenario to complex living organisms entails drastic dilution of the active cargo, nuclease degradation, low-pH conditions in the stomach, mucociliary clearance, inefficient cell-membrane penetration, endosomal-lysosomal escape, and additional physical barriers (43). The considerable variability across organisms and diets is precisely what makes this statement a reproducibility concern. It is notable, though, that plant miRNAs have been detected in digestive waste, suggesting that, once shielded by EV encapsulation, exogenous dietary miRNAs can partially persist in the human body, resisting degradation (65).

It is likewise worth emphasizing that the evidence assembled throughout this review spans different evidence dimensions, including computational, correlational, cellular, animal, and human observational. Therefore, each should be interpreted according to the type of evidence it provides. While certain findings merely point to a correlation and therefore warrant more exhaustive evaluation across multiple independent axes, others provide more direct mechanistic or functional support (Figure 3). It is equally important to distinguish evidence obtained after normal dietary consumption from studies using purified food-derived extracellular vesicles or synthetic miRNA mimics, as these experimental approaches may expose cells or animals to concentrations that do not reflect physiological dietary intake. Neither category, however, is exempt from further validation. In this way, only through multiple independent investigations can a definitive claim be reached and this controversy ultimately clarified.

**Figure 3:**
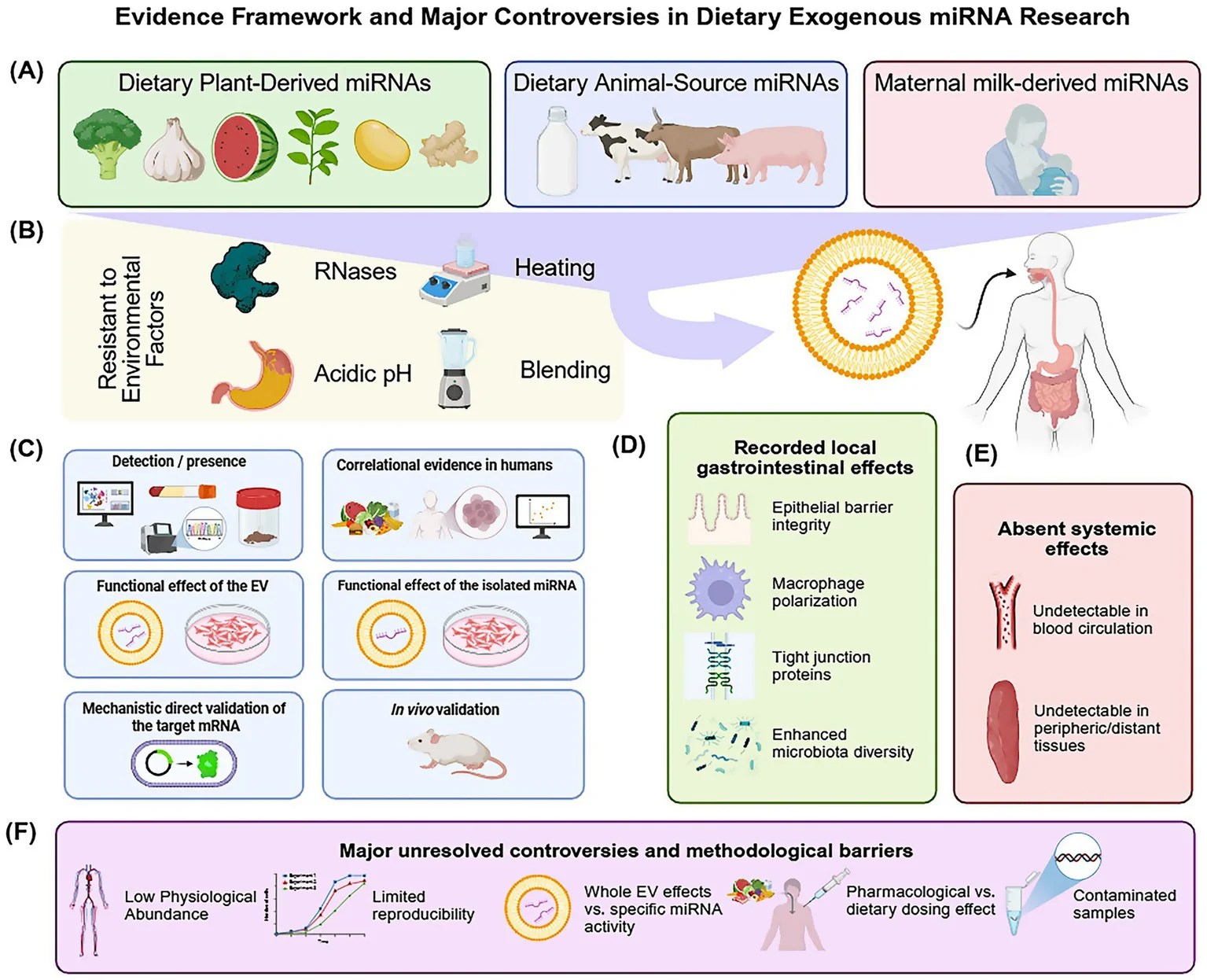
Visual framework of the key concepts of the current research on dietary exogenous miRNAs. **(A)** Principal 3 origins of dietary exogenous miRNAs discussed in this article. The plant-derived illustrations are representative. **(B)** The extracellular vesicles that encapsulate the miRNAs and can be orally ingested protect the miRNA cargo from RNases, heating, blending, and acidic environments. **(C)** Evidence framework of the current research on dietary exogenous miRNAs. Evidence is organized into complementary, non-sequential dimensions: detection/presence of miRNAs in feces and/or serum implying gastrointestinal stability; correlational evidence in humans through computational associations; functional causal effects of intact EVs *in vitro*; functional causal effects of isolated dietary miRNAs *in vitro*; mechanistic direct target validation of miRNA–mRNA interactions; and *in vivo* validation with physiological exposure relevance. **(D)** The known local gastrointestinal effects of dietary exogenous miRNAs include conservation of the epithelial barrier integrity, M2 polarization, restoration of tight junction proteins, and a balanced diversity of microbiota. **(E)** Definitive systemic effects of orally consumed miRNAs remain controversial and inconsistent. **(F)** The main unresolved controversies on this topic include a low or even absent physiological detection of miRNAs, limited reproducibility of experiments across different evidence dimensions, discernment of the effects of a single miRNA and the raw and heavily loaded EVs, scope of orally consumed miRNAs via normal diet and injection of specific purified miRNAs, and potentially contaminated samples when screening for miRNAs in the human body. Image created in BioRender.com.

EVs appear to be a particularly promising vehicle by which dietary exogenous miRNAs can influence host tissues, at least at a local level. Zhu et al. (55), Liu et al. (45), Yan et al. (50), Lu et al. (60), Luo et al. (53), Shi et al. (56), Xu et al. (58), and Wang et al. (54) have provided evidence that food-derived miRNAs administered orally to mice aided in the preservation of a good intestinal architecture and in the alleviation of symptoms related to gastrointestinal diseases. Accordingly, it was defined throughout this manuscript that a dietary exogenous miRNA-rich diet can exert defined effects at the tissue level, particularly within the gut lumen. Nonetheless, because many studies administer intact extracellular vesicles rather than isolated miRNAs; therefore, the observed phenotypes cannot necessarily be attributed to the miRNA cargo itself. In earlier years, when such evidence remained scarce and negative results predominated, the scientific community leaned toward skepticism and demanded positive results from studies capable of ruling out contamination and addressing the kinetics and mechanisms of dietary miRNA absorption, distribution, and clearance (66). Now, the increasingly consistent replication of these effects, exemplified throughout this review, points toward a more promising therapeutic application for alleviating colitis, inflammatory bowel disease, and even colorectal cancer.

No account of intestinal dietary exogenous miRNA activity, however, is complete without considering gut bacteria. Zhu et al. (55), Xu et al. (58), and Wang et al. (54) have consistently reported that dietary miRNAs can have prebiotic-like effects, modulating abundance ratios, diversity, and even strain type, thereby ameliorating intestinal homeostasis and colon health. The precise mechanism by which exogenous miRNAs achieve sufficient bacterial bioavailability to exert such regulatory control remains to be elucidated. In fact, changes in microbial community composition following whole-vesicle administration should also be interpreted with caution, as they may reflect indirect effects of improvements in intestinal inflammation or barrier function rather than direct bacterial uptake or miRNA targeting. This represents another frontier for studying the clinical potential of orally ingested exogenous miRNAs. This area of research is a relatively young, rapidly expanding field, offering broad scope for deeper mechanistic investigation (67).

Exploring this novel path, where contradictory evidence exists regarding the diet-host-bacteria interaction, is, in many respects, precisely how science advances. Each novel discovery initially raises more questions, and replication is hard to achieve. Yet, as understanding deepens, the path forward grows progressively clearer. Appropriately, new findings are questioned and subjected to rigorous scrutiny, with the aim of resolving the doubts and gaps in the research. It would be unreasonable to expect a definitive, unequivocal statement on whether exogenous dietary miRNAs influence host health soon after the first result was published. Eventually, interdisciplinary efforts spanning microbiology, bioinformatics, transcriptomics, systems biology, and proteomics will progressively refine this body of knowledge to prove or argumentatively refute the causal role of dietary exogenous miRNAs in gastrointestinal homeostasis. Connecting the current gaps and disentangling the existing discrepancies will, in due course, reveal the exact contribution of dietary exogenous miRNAs in the maintenance of gastrointestinal health, opening the way to explore their potential in disease detection, prevention, and even therapeutic interventions.

## 5 Conclusion

Considerable progress has been made toward elucidating the behavior of EV-encapsulated dietary exogenous miRNAs within the gastrointestinal environment; nevertheless, the evidence assembled to date remains contradictory, inconclusive, and only partially understood, leaving a substantial gap for further inquiry in this emerging field. The evidence presented in this article includes well-documented instances of exogenous dietary miRNAs detected both in the gastrointestinal tract and in digestive waste products, several of which have been shown to modulate the growth and diversity of intestinal bacteria. But more significantly, they have provided evidence of their capacity to regulate the expression of both bacterial and host genes, translating into measurable amelioration of intestinal cell morphology and inflammatory symptomatology. The documented local effects within the gut therefore are presented as more biologically plausible than widespread systemic gene regulation. Notwithstanding this, the reproducibility and extrapolation of these findings to human clinical trials remain unresolved at present. Achieving this step requires additional rigorous studies. We contend that this body of evidence could mark the incipient stages of a shift in which orally administered miRNAs warrant further investigation as a potential avenue for intestinal disease research.
